# Inactivation of spores by electric arcs

**DOI:** 10.1186/s12866-016-0764-x

**Published:** 2016-07-12

**Authors:** Flavien Pillet, Igor Marjanovič, Matej Reberšek, Damijan Miklavčič, Marie-Pierre Rols, Tadej Kotnik

**Affiliations:** CNRS, IPBS (Institut de Pharmacologie et de Biologie Structurale), 205 Route de Narbonne BP64182, F-31077 Toulouse, France; Université de Toulouse, UPS, IPBS, F-31077 Toulouse, France; Department of Biomedical Engineering, Faculty of Electrical Engineering, University of Ljubljana, Tržaška 25, SI-1000 Ljubljana, Slovenia

**Keywords:** Electric arc discharges, Bacterial spore eradication

## Abstract

**Background:**

In the context of spore contamination involved in bio-terrorism and food preservation, the development of new techniques for spore inactivation is an important challenge.

**Results:**

Here, a successful application of electric arc discharges resulting in spore death was reported. Two types of electric arcs were compared, different with respect to their durations. The discharges with 0.5 μs duration induced a small inactivation area of 0.6 % of surface treated around their point of entry into the sample, while those with 20 μs duration induced a much larger inactivation area from 7 to 55 % of surface treated roughly proportional to the number of discharges delivered. In particular, 50 discharges of 20 μs duration induced inactivation in more than 55% of surface treated at an inactivation rate above 3.6 log10.

**Conclusions:**

These results are promising and warrant developing electric arcing as a novel method for spore inactivation.

**Electronic supplementary material:**

The online version of this article (doi:10.1186/s12866-016-0764-x) contains supplementary material, which is available to authorized users.

## Background

Bacterial spores are one of the most resilient life forms known, exceptionally resistant to chemical, environmental and physical stresses [1]. Spores can survive in dormant phase in extreme conditions, even in space, [2] and can hence cause contamination during interplanetary missions [3]. Furthermore, spores can remain viable even during geological time spans; for instance, spores of *Bacillus subtilis* were recovered and revived from abdominal contents of extinct bees trapped in amber fossilized 25 to 40 million years ago [4]. Such resilience and endurance of bacterial spores is explained by a highly dehydrated structure of their core that includes their genomic material and ribosomes, and protection of this core by a multilayer envelope, consisting of a highly impermeable inner membrane, a temperature resistant peptidoglycan cortex, an outer membrane, and chemically resistant protein coat [5]. This resilience poses considerable obstacles in inactivation of pathogenic spores, which are related to large number of different diseases. Thus, bacterial spores can cause respiratory infection (e.g. *Bacillus anthracis* acting as the etiologic agent of anthrax [6]), food contamination (e.g. by *Clostridium botulinum* causing botulism [7]), and fatal paralytic illness (e.g. *Clostridium difficile* involved in infectious diarrhea [8]).

Within this context of high noxiousness and resistance to extreme conditions, efficient methods for inactivation of pathogenic spores are of utmost importance. Sterilization by heating above 100 °C is classically used and efficient for food preservation [9], but cannot be used for decontamination of thermally sensitive materials. Two alternatives are gamma irradiation [10] and exposure to ethylene oxide [11], which are efficient, but expensive and often also harmful to the matter being decontaminated.

Another approach is to expose the material to high-voltage atmospheric cold plasma (HVACP). Plasma results in spore inactivation by generation of antimicrobial agents including reactive oxygen species (ROS), ultraviolet light (UV) and charged particles [12–14]. An alternative is to deliver the electric pulses through an air gap, thus generating an arc discharge; in this case, there are several effects acting simultaneously, the sample is exposed to ultraviolet light [15], as well as to an acoustic shockwave [16], in which the brief surge of mechanical pressure can also cause cavitation [17]. This multiple-effect approach was first described in 1962 for inactivation of bacteria in water in their vegetative state [18], but it has apparently not yet been tested on their much more resilient spores.

Here, this paper describe for the first time a successful application of electric arc discharges resulting in inactivation of spores of *Bacillus pumilus*, a non-pathogen model of *Bacillus anthracis*. This inactivation was performed by delivering an arc discharge through an air gap into the sample of spores deposited on agar, with the exposure system developed and described previously by Marjanovič and Kotnik [19], yet with a more powerful discharge generator developed subsequently. The spore inactivation was compared with two types of electrical discharges: 0.5 μs and 20 μs electric arcs, of which particularly the latter were accompanied by an intense light emission and acoustic shockwave.

## Methods

### Preparation of spores

Spores of *Bacillus pumilus* (ATCC 27142) were produced after 5 days culture at 37 °C in Difco sporulation medium (DSM), as previously described by Schaeffer et al [20]. The remaining vegetative bacteria were inactivated by pasteurization (80 °C for 20 min) and lysed by 1 h exposure at 37 °C in a lysozyme solution (50 μg/ml of lysozyme in 50 mM Tris-HCl pH6.2). The spores were centrifuged (5 min, 10,000 g) and washed 1 time in deionized water, 1 time in SDS 0.02 % and 3 times in deionized water. For each experiment, 1 ml of spore solution containing about 2.10^7^ spores/ml was added and spread in a Petri dish with a 90 mm diameter (Sterilin plate, Thermo Scientific, UK). The overflow was removed (900 μl) and the Petri dish was dried 15 min prior to experiment. The number of spores estimate was 2.10^6^ by Petri dish.

### Exposure to electric arcs

**Fig. 1:**
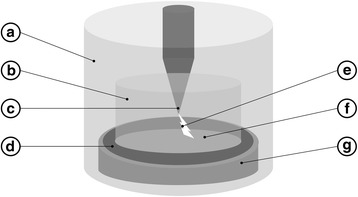
Schematic representation of the exposure system developed by Marjanovič and Kotnik [19]. The system is enclosed by a transparent plexi-glass tube (a). An internal container also made of plexi glass (b) prevents the electric arc from short-circuiting the conical emitting electrode (c) and the ring-shaped receiving electrode (d). The emitting electrode is in the air above the sample, while the receiving electrode is in direct contact with the outer edge of the disk-shaped sample. An electric arc is illustrated (e) as exiting downwards from the emitting electrode into the sample containing the spores (f) deposited on the Petri dish (g)

The exposure system is shown schematically in Fig. 1, and was previously described in detail by Marjanovič and Kotnik in 2013 [19]. Before exposure, the ring electrode was placed in contact at the periphery of the Petri dish. Electric arcs were generated between the emitting tip electrode and the ring electrode. The air gap between the tip electrode and the agar surface was 1 mm. Two generators were used with different time of electric arc exposure: a Taser gun (Great Power 750,000, Great Power Co., South Korea) delivering ~0.5 μs electric arcs of ~12 kV at ~100 A, and a custom-made generator built at the Faculty of Electrical Engineering, University of Ljubljana delivering 20 μs electric arcs of ~5 kV at ~50 A [21].

### Quantification of inactivation

The spore density in control condition was evaluated by colony counting from successive dilutions of spores spread and incubated overnight at 37 °C (Additional file 1). First, the inactivation areas were measured with the software ImageJ 1.46r (National Institutes of Health, USA). Then, the inactivation rates in the areas exposed to electric arcs were calculated as the ratio between the spore density under control conditions and the spore density in the areas of exposure. The inactivation area and the inactivation rate were calculated from 3 independent experiments.

## Results

### Inactivation of spores by 0.5 μs electric arcs

**Fig. 2:**
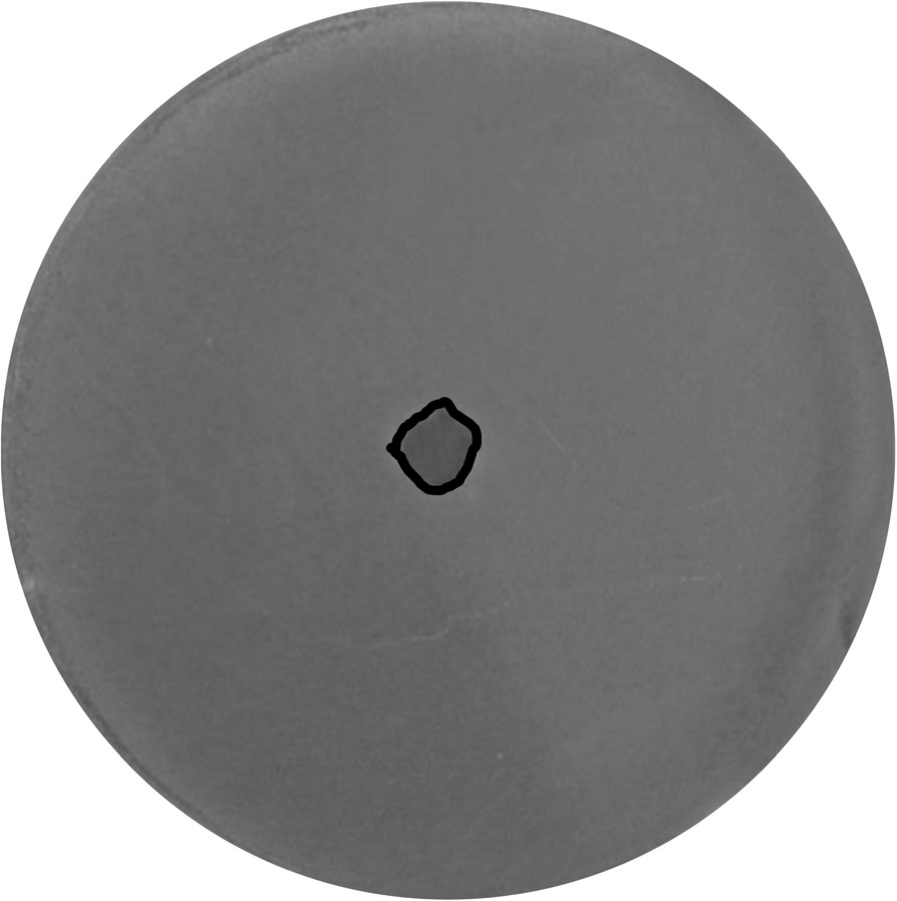
Spore inactivation with ~20 electric arc discharges of ~0.5 μs duration. The inactivation area is outlined by the black curve

The Fig. 2 show the effects of delivering ~20 electric arc discharges, each with an ~0.5 μs duration, delivered in intervals of ~300 ms (the durations of the discharges and the intervals between them as delivered by the Taser gun, see Exposure to electric arcs, were neither adjustable nor highly reproducible, but the monitoring of the exposures on the oscilloscope assured that in no experiment their actual values differed from those stated above by more than 10 %).

**Fig. 3:**
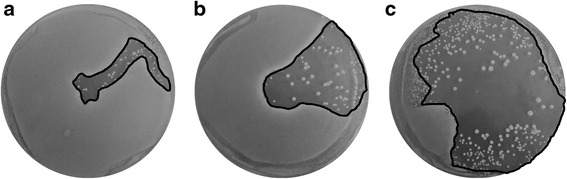
Spore inactivation with 1 (**a**)**,** 10 (**b**), and 50 (**c**) electric arc discharges of 20 μs duration. The inactivation area is outlined by the black curve

In these exposures, the arc always descended from the tip of the conical emitting electrode downwards and entered centrally into the sample, with the electric current then dissipating roughly radially outwards through the sample to the ring-shaped receiving electrode (for a photographic example, see e.g. Fig. 3 in Marjanovič and Kotnik [19]). In this manner, the current density and induced electric field decreased roughly inversely to the distance from the point of the arc entry into the sample. As a consequence, the area of spore inactivation caused by the exposure was roughly centered at the Petri dish midpoint, and it was rather small, as only ~0.65 % of the total Petri dish surface was subject to inactivation, but inactivation there was complete, with no detectable colony within this area (Fig. 2).

### Inactivation of spores by 20 μs electric arcs

The Fig. 3 show the effects of delivering a varying number of electric arc discharges, from 1 to 50, each with a 20 μs duration, delivered in intervals of ~3 s.

In these exposures, the arc also initially descended from the tip of the conical emitting electrode downwards into the sample, but then tended to tilt gradually but increasingly sideways, so that after 8–12 μs, it created a direct diagonal connection between the tip of the conical emitting electrode and the ring-shaped receiving electrode, thus largely proceeding above the sample.

For a single 20 μs electric arc, the path of the arc from its initial almost vertical descent into the sample, along its gradual lateral shift towards the receiving electrode at the sample’s edge, to its formation of a direct connection with this electrode, is clearly visible in Fig. 3a, with the area of inactivation formed along this path and its immediate vicinity.

With an increasing number of arc discharges delivered, consecutive arcs tended to form plasma channels in directions proximate to the direction of the first discharge (as seen in Fig. 3b for the case of 10 discharges), yet they gradually scattered, eventually covering most of the sample (as seen in Fig. 3c for the case of 50 discharges). Thus, due to random variations in the paths of consecutive arcs between the electrodes, the inactivation area gradually increased with the number of discharges delivered, reaching 7 % for 1 arc, 27 % for 10 arcs and 55 % for 50 arcs as outlined by the black curve in the three panels of Fig. 3, and stated quantitatively in Table 1. However, the inactivation rate was similar in the area inactivated with a spore inactivation about 3.6 log10. Outside this area, there was no detectable inactivation.

**Table 1:** Calculation of spore inactivation with 20 μs electrics arcs

	Inactivation area (%)	Inactivation rate (log_10_)
1 arc	7 ± 0.6	3.4 ± 0.14
10 arcs	27 ± 4.5	3.4 ± 0.32
50 arcs	55 ± 12.0	3.6 ± 0.36

## Discussion

The results presented above demonstrate that electric arc discharges can cause substantial inactivation of spores of *Bacillus pumilus*. As efficient methods for spore inactivation are lacking, yet of utmost importance, these results are promising and warrant further investigation. Still, much further work is needed before electric arcing could become established as a method for spore inactivation.

First, the duration of electric discharges is directly correlated with the arc’s path. For the discharge duration of 0.5 μs, the inactivation area is limited to a small area centered at the midpoint of the Petri dish. However, as the discharge duration increases, so does the path length of the arc, extending gradually outwards, and for discharge durations exceeding 10 μs, the arc reaches the outer ring-shaped electrode. Consequently, the inactivation area is also substantially larger with an 20 μs electric arc, and additionally increases, as explained above, when more than one discharge is applied, as the paths of consecutive arcs are not identical, but vary randomly. Second, our experiments have been performed in 90-mm Petri dishes, while for applications of spore inactivation, the interest both industrially and clinically lies on much larger areas. Testing of electric arcing’s efficiency on such scales will require correspondingly upscaled exposure systems and generators, which also raises the problem of operating safety, which at the scale used here is much less acute.

Finally, as mentioned in the introduction, unlike plasma discharges, where exposure is to UV and ROS [13], arc discharges also expose the sample to mechanical (acoustic) pressure wave; very close to the arc’s point of entry from air into the sample, perhaps in the closest few square millimeters at the sample’s very surface, also the highly elevated temperature of the locally plasmified air certainly plays a role. The relative contributions of these effects to the final rate of inactivation have not been evaluated here, and for practical applications of inactivation are also not of particular relevance, yet for fundamental understanding of the basic mechanisms involved they will have to be investigated. This will likely require intricate modifications and upgrades to the experimental apparatus used here, but it’s certain that such a reductionist study is to a large extent feasible.

## Conclusion

We evaluated for the first time, the inactivation of bacterial spores by electric arcs. The best results were obtained with 20 μs electric arcs at 5 kV, with an inactivation rate exceeding 3 log_10_. Additional physical and chemical phenomena probably affected the inactivation, such as shock wave, ionization, UV radiation etc. Supplementary studies should be done to discriminate the impact of each parameter.

## Additional file

Additional file 1: Determination of density of untreated spores. Successive spore dilutions of 1 hundred were shown after spread and incubation overnight at 37°C. A bacterial mat was observed for the 1/1 dilution (a). A very high density of colony was visualized in the 1/100 dilution (b). 222 colony was counted in the 1/10 000 dilution (c). With a petri dish surface of 63.6 cm², we calculated a spore density of 3.5 104 spore by cm² in the 1/1 dilution. (DOCX 2.59 MB)
