# Supplementary material for: Inactivation of spores by electric arcs
Source: BMC Microbiol. 2016 Jul 12;16:148. doi: 10.1186/s12866-016-0764-x (PMC4942915; doi:10.1186/s12866-016-0764-x)
Supplement: Additional file 1: — Determination of density of untreated spores. Successive spore dilutions of 1 hundred were shown after spread and incubation overnight at 37°C. A bacterial mat was observed for the 1/1 dilution (a). A very high density of colony was visualized in the 1/100 dilution (b). 222 colony was counted in the 1/10 000 dilution (c). With a petri dish surface of 63.6 cm², we calculated a spore density of 3.5 104 spore by cm² in the 1/1 dilution. (DOCX 2.59 MB) [file 12866_2016_764_MOESM1_ESM.docx]

**Additional file 1: Determination of density of untreated spores**. Successive spore dilutions of 1 hundred were shown after spread and incubation overnight at 37°C. A bacterial mat was observed for the 1/1 dilution (a). A very high density of colony was visualized in the 1/100 dilution (b). 222 colony was counted in the 1/10 000 dilution (c). With a petri dish surface of 63.6 cm², we calculated a spore density of 3.5 10^4^ spore by cm² in the 1/1 dilution.
